# COVID-19 vaccines: rapid development, implications, challenges and future prospects

**DOI:** 10.1007/s13577-021-00512-4

**Published:** 2021-03-07

**Authors:** Shivaji Kashte, Arvind Gulbake, Saadiq F. El-Amin III, Ashim Gupta

**Affiliations:** 1 Department of Stem Cell and Regenerative Medicine, Center for Interdisciplinary Research, D.Y. Patil Education Society (Institution Deemed To Be University), Kolhapur, Maharashtra 416006 India; 2 Dehradun Institute of Technology (DIT) University, Dehradun, Uttarakhand 248009 India; 3 El-Amin Orthopaedic and Sports Medicine Institute, Lawrenceville, GA 30043 USA; 4 BioIntegrate, Lawrenceville, GA 30043 USA; 5 South Texas Orthopaedic Research Institute, Laredo, TX 78045 USA; 6 Veterans in Pain, Valencia, CA 91354 USA; 7 Future Biologics, Lawrenceville, GA 30043 USA

**Keywords:** SARS-Cov-2, COVID-19, COVID-19 vaccine, Vaccine hesitancy, Emergency use authorization

## Abstract

COVID-19 has affected millions of people and put an unparalleled burden on healthcare systems as well as economies throughout the world. Currently, there is no decisive therapy for COVID-19 or related complications. The only hope to mitigate this pandemic is through vaccines. The COVID-19 vaccines are being developed rapidly, compared to traditional vaccines, and are being approved via Emergency Use Authorization (EUA) worldwide. So far, there are 232 vaccine candidates. One hundred and seventy-two are in preclinical development and 60 in clinical development, of which 9 are approved under EUA by different countries. This includes the United Kingdom (UK), United States of America (USA), Canada, Russia, China, and India. Distributing vaccination to all, with a safe and efficacious vaccine is the leading priority for all nations to combat this COVID-19 pandemic. However, the current accelerated process of COVID-19 vaccine development and EUA has many unanswered questions. In addition, the change in strain of SARS-CoV-2 in UK and South Africa, and its increasing spread across the world have raised more challenges, both for the vaccine developers as well as the governments across the world. In this review, we have discussed the different type of vaccines with examples of COVID-19 vaccines, their rapid development compared to the traditional vaccine, associated challenges, and future prospects.

## Background

Severe acute respiratory syndrome coronavirus 2 (SARS-Cov-2) infections and the resulting diseases, coronavirus disease 2019 (COVID-19) have spread to millions of people worldwide. The World Health Organization (WHO) declared the COVID-19, a pandemic in March 2020 [1]. The SARS-CoV-2 has affected over 105 million people and has claimed over 2.29 million lives worldwide, as of February 5, 2021. The most affected countries have been the United States of America, with over 26.7 million cases and 456,000 deaths, and India, with over 10.8 million cases and 155,000 deaths as of February 5, 2021 [2]. COVID-19 has negatively impacted the health and lifestyle of people as well as the economy throughout the world [3]. An intensive search for an effective drug against the SARS-CoV-2 did not lead to any breakthrough candidates. The drugs like Hydroxychloroquine and Remdesivir were advocated as desperate measures based on contradictory and inconclusive studies and have significantly failed to combat the pandemic [4]. As the number of COVID-19 patients continues to increase, detecting, assessing, and interpreting the immune response to SARS-CoV-2 infection becomes essential. Multiple vaccine candidates are under development but safe and effective vaccines against COVID-19 are urgently needed to combat escalating cases and deaths worldwide. These vaccine candidates need to be manufactured as soon as possible and made available to all countries and populations affected by the pandemic at an affordable price. A vaccine has the ability to induce herd immunity in societies, which can decrease the occurrence of the disease, block transmission, and reduce the social and economic burden of the disease.

On December 2, 2020, United Kingdom (UK) became the first country to approve the COVID-19 vaccine, BNT162, developed by Pfizer and BioNTech via Emergency Use Authorization (EUA). WHO approved BNT162 for emergency use on December 31, 2020 to allow for easier global manufacturing and distribution. Similar EUA processes were adapted by several countries including, United States, Canada, Russia, China, and India to approve different COVID-19 vaccine candidates (CVCs) and the list is growing. There are a total of 232 vaccine candidates at various stages of development, of which 172 are in preclinical development, 60 are in clinical development, and 9 are approved under EUA by different countries (Tables 1 and 2) [5]. Despite the rollout of these vaccines under EUA, several questions need to be answered. How are these vaccines developed so rapidly? Are these vaccines safe and efficacious? How long the efficacy will last? What are potential threats? What are challenges? Are they effective against changing strains of virus? In this review, we will discuss and emphasize the vaccines approved via EUA and the ones that have entered Phase III clinical trials and have demonstrated the potential to be approved.

**Table 1:** COVID-19 Vaccine candidates that are in clinical phase and approved under Emergency Use Authorization by different countries

COVID-19 Vaccine developer/manufacturer	Vaccine candidate name	Type of vaccine	No. of doses; duration; route of administration	Claimed efficacy	Clinical stage					Authorization/Approved
					Phase 1	Phase 1/2	Phase 2	Phase 2/3	Phase 3	
Pfizer, BioNTech	BNT162	3 LNP-mRNAs	2; 0, 28 days; IM	95%	NCT04523571	ChiCTR2000034825 NCT04537949 NCT04380701 EUCTR2020-003267-26-DE	NCT04588480 NCT04649021	NCT04368728	–	UK, US, Canada, Mexico, Bahrain
Moderna, Kaiser Permanente Washington Health Research Institute	mRNA-1273	LNP-encapsulated mRNA	2; 0, 28 days; IM	94%	NCT04283461	–	NCT04405076	NCT04649151	NCT04470427	US
University of Oxford/AstraZeneca	AZD1222	Non- Replicating Viral Vector ChAdOx1-S	2; 0, 28 days; IM	70%	PACTR202005681895696	PACTR202006922165132 NCT04568031 NCT04444674 NCT04324606	–	NCT04400838 EUCTR2020-001228-32-GB CTRI/2020/08/027170	ISRCTN89951424 NCT04516746 NCT04540393 NCT04536051	UK
Gamaleya Research Institute, Acellena Contract Drug Research and Development	Sputnik V	Non-replicating viral vector	2; 0, 21 days; IM	92%	–	NCT04436471 NCT04437875	NCT04587219	NCT04640233	NCT04530396 NCT04564716 NCT04642339 NCT04656613	Russia
Federal Budgetary Research Institution State Research Center of Virology and Biotechnology	EpiVacCorona	Peptide vaccine	–	–	–	NCT04527575	–	–	–	Russia
Sinovac	CoronaVac	Inactivated Vaccine (formalin with alum adjuvant)	2; 0, 14 days; IM	–	–	NCT04383574 NCT04352608 NCT04551547	–		NCT04456595 NCT04508075 NCT04582344 NCT04617483 NCT04651790	China
Beijing Institute of Biological Products/Sinopharm	BBIBP-CorV	Inactivated	2; 0, 21 days; IM	86%	–	ChiCTR2000032459	–		ChiCTR2000034780 NCT04560881 NCT04510207	China, United Arab Emirates
Wuhan Institute of Biological Products, China, National Pharmaceutical Group (Sinopharm)	Not given yet	Inactivated	2; 0, 21 days; IM	–	–	ChiCTR2000031809	–		ChiCTR2000034780 ChiCTR2000039000 NCT04612972 NCT04510207	China

**Table 2:** Potential COVID-19 vaccine candidates that are in the clinical phase

COVID-19 vaccine developer/manufacturer	Type of vaccine	Number of doses, timing of doses, route of administration	Clinical stage				
			Phase 1	Phase 1/2	Phase 2	Phase 2/3	Phase 3
Bharat Biotech	Whole-Virion Inactivated	2; 0,28 days; IM	–	NCT04471519 CTRI/2020/07/026300 CTRI/2020/09/027674	–	–	NCT04641481 CTRI/2020/11/028976
CanSino Biological Inc./Beijing Institute of Biotechnology	Non- Replicating Viral Vector Adenovirus Type 5 Vecto	1; IM	ChiCTR2000030906 NCT04568811 NCT04313127 NCT04552366	NCT04398147	ChiCTR2000031781 NCT04566770 NCT04341389	–	NCT04526990 NCT04540419
Janssen Pharmaceutical Companies	Non- Replicating Viral Vector Adenovirus Type 26 vector	1; 0 days 2; 0, 56 days; IM	NCT04509947	NCT04436276	EUCTR2020-002584-63-DE NCT04535453	–	NCT04505722 NCT04614948
Novavax	Full length recombinant SARS CoV-2 glycoprotein nanoparticle vaccine adjuvanted with Matrix M	2; 0, 21 days; IM	–	NCT04368988	NCT04533399	–	NCT04611802 NCT04583995 EUCTR2020-004123-16-GB
Anhui Zhifei Longcom Biopharmaceutical/Institute of Microbiology, Chinese Academy of Sciences	Adjuvanted recombinant protein (RBD-Dimer) expressed in CHO cells	3; 0, 28, 56 days; IM	NCT04445194 NCT04636333 ChiCTR2000035691	NCT04550351	NCT04466085	–	ChiCTR2000040153 NCT04646590
Medicago Inc	Plant-derived VLP adjuvanted with AS03. 0, 21 days 0, 21 days	2; 0, 21 days; IM	NCT04450004	–	NCT04662697	NCT04636697	–
INOVIO Pharmaceuticals/ International Vaccine Institute	DNA plasmid vaccine with electroporation	2; 0, 28 days; ID	NCT04336410	NCT04447781	ChiCTR2000040146	NCT04642638	–
Jiangsu Provincial Center for Disease Prevention and Control	Replicating Viral Vector Intranasal flu-based-RBD	1; IN	ChiCTR2000037782	–	ChiCTR2000039715	–	–
West China Hospital, Sichuan University	RBD (baculovirus production expressed in Sf9 cells)	2or3; 0, 28 days and 0,14, 28 days; IM	ChiCTR2000037518 NCT04530656	–	ChiCTR2000039994 NCT04640402	–	–
Curevac	mRNA	2; 0, 28 days; IM	NCT04449276	PER-054-20	NCT04515147	NCT04652102	EUCTR2020-004,066–19 NCT04674189
Institute of Medical Biology, Chinese Academy of Medical Sciences	Inactivated	2; 0, 28 days; IM		NCT04470609 NCT04412538	–	–	NCT04659239
Research Institute for Biological Safety Problems, Rep of Kazakhstan	Inactivated	0, 21 days	–	NCT04530357	–	–	–
Shenzhen Kangtai Biological Products Co., Ltd	Inactivated	2; IM	ChiCTR2000038804	–	ChiCTR2000039462	–	–
Osaka University/ AnGes/ Takara Bio	DNA plasmid vaccine + Adjuvant	2; 0, 14 days; IM	–	NCT04463472 NCT04527081	–	NCT04655625	–
Cadila Healthcare Limited	DNA plasmid vaccine	3; 0, 28, 56 days; ID	–	CTRI/2020/07/026352	–	–	–
Genexine Consortium	DNA Vaccine (GX-19)	2; 0, 28 days; IM	–	NCT04445389	–	–	–
Kentucky Bioprocessing, Inc	RBD-based	2; 0, 21 days; IM	–	NCT04473690	–	–	–
Sanofi Pasteur/GSK	S protein (baculovirus production)	2; 0, 21 days; IM	–	NCT04537208	–	–	–
Israel Institute for Biological Research	Replicating Viral Vector VSV-S	1; IM	NCT04608305	–	–	–	–
Arcturus/Duke-NUS	mRNA	IM	–	NCT04480957	NCT04668339	–	–
Serum Institute of India/ Accelagen Pty	RBD-HBsAg VLPs	2; 0, 28 days; IM	–	ACTRN12620000817943	–	–	–
Symvivo	bacTRL-Spike	1; Oral	NCT04334980	–	–	–	–
Providence Health & Services	electroporated S protein plasmid DNA vaccine with or without the combination of electroporated IL-12p70 plasmid	2; 0, 14 days; ID	NCT04627675	–	–	–	–
Codagenix/Serum Institute of India	Codon deoptimized live attenuated vaccines	1 or 2; 0 or 0,28 days; IN	NCT04619628	–	–	–	–
ImmunityBio, Inc. & NantKwest Inc	hAd5 S + N 2nd Generation Human Adenovirus Type 5 Vector (hAd5) Spike (S) + Nucleocapsid (N)	1; 0 day; Oral	NCT04591717	–	–		–
ReiThera/LEUKOCARE/Univercells	Non-Replicating Viral Vector Simian Adenovirus (GRAd) encoding S	1; IM	NCT04528641	–	–	–	–
Vaxart	Non-Replicating Viral Vector Ad5 adjuvanted Oral Vaccine platform	2; 0, 28 days; Oral	NCT04563702	–	–	–	–
Ludwig-Maximilians—University of Munich	Non-Replicating Viral Vector MVA-SARS-2-S	2; 0, 28 days; IM	NCT04569383	–	–	–	–
City of Hope Medical Center/National Cancer Institute, USA	SARS-CoV-2 S and NP genes inserted into a Replicating Viral Vector sMVA	2; 0, 28 days; IM	NCT04639466	–	–	–	–
Clover Biopharmaceuticals Inc./GSK/Dynavax	Native like Trimeric subunit Spike Protein vaccine	2; 0, 21 days;IM	NCT04405908	–	–	NCT04672395	–
Vaxine Pty Ltd/Medytox	Recombinant spike protein with Advax™ adjuvant	1; IM	NCT04453852	–	–	–	–
Medigen Vaccine Biologics Corporation/NIAID/Dynavax	S-2P protein + CpG 1018	2; 0, 28 days	NCT04487210	–	–	–	–
Instituto Finlay de Vacunas, Cuba	RBD + Adjuvant	2; 0, 28 days; IM	RPCEC00000338 RPCEC00000340	RPCEC00000332	–		–
University Hospital Tuebingen	SARS-CoV-2 HLA-DR peptides	1; SC	NCT04546841	–	–	–	–
COVAXX / United Biomedical Inc. Asia	Multitope peptide-based S1-RBDprotein vaccine	2; 0, 28 days; IM	NCT04545749	–	–	NCT04683224	–
Institute Pasteur/Themis/Univ. of Pittsburg CVR/Merck Sharp & Dohme	Replicating Measles-vector based	1–2; 0, 28 days; IM	NCT04497298 NCT04569786	CT04498247	–	–	–
Imperial College London	LNP-nCoVsaRNA	2; IM	ISRCTN17072692	–	–	–	–
Shulan (Hangzhou) Hospital + Center for Disease Control and Prevention of Guangxi Zhuang Autonomous Region	mRNA	2; 0, 14 or 0, 28 days; IM	ChiCTR2000034112 ChiCTR2000039212	–	–	–	–
Barbara Carlson, University of Oklahoma	Protein subunit Zoster Vaccine Recombinant, Adjuvanted	2; 0, 60 day; IM	NCT04523246	–	–	–	–
Adimmune Corporation	AdimrSC-2f (recombinant RBD ± Aluminium)	-	NCT04522089	–	–	–	–
Entos Pharmaceuticals Inc	DNA based vaccine Covigenix VAX-001	2; 0, 14 days; IM	NCT04591184	–	–	–	–
Chulalongkorn University	ChulaCov19 mRNA vaccine	2; 0, 21 days; IM	NCT04566276	–	–	–	–
Aivita Biomedical, Inc	Viral vector (Replicating) + APC	1; 0 day; IM	–	NCT04386252	–	–	–
Center for Genetic Engineering and Biotechnology (CIGB)	CIGB-669 (RBD + AgnHB)	3; 0, 14, 28 or 0, 28, 56 day; IN	–	RPCEC00000345	–	–	–
Center for Genetic Engineering and Biotechnology (CIGB)	CIGB-66 (RBD + aluminium hydroxide)	3; 0, 14, 28 or 0, 28, 56 day; IN	–	RPCEC00000346	–	–	–
Instituto Finlay de Vacunas	FINLAY-FR anti-SARS-CoV-2 Vaccine (RBD + adjuvant)	2; 0, 28 day; IM	RPCEC00000338 RPCEC00000340	RPCEC00000332	RPCEC00000347	-	-
Valneva, National Institute for Health Research, United Kingdom	Inactivated Virus VLA2001	2; 0, 21 day; IM	–	NCT04671017	–	–	–
Biological ELimited	Protein subunit BECOV2	2; 0, 28 day; IM	–	CTRI/2020/11/029032	–	–	–
Cellid Co., Ltd	Viral vector (Replicating) AdCLD-CoV19	IM	–	NCT04666012	–	–	–
GeneOne Life Science, Inc	DNA based vaccine GLS-5310	2; 0, 56 or 0, 84 day; ID	–	NCT04673149	–	–	–
Nanogen Pharmaceutical Biotechnology*	Recombinant Sars-CoV-2 Spike protein, Aluminium adjuvanted	2; 0, 21 day; IM	–	NCT04683484	–	–	–
Shionogi*	Recombinant protein vaccine S-268019 (using Baculovirus expression vector system)	2; 0, 21 day; IM	–	jRCT2051200092	–	–	–

## What is a vaccine?

“Vaccines are biologics that provide active adaptive immunity against specific diseases” [5]. Vaccine development involves utilizing the microorganisms responsible for the disease either in the killed or attenuated form, or it involves the use of microorganisms’ toxins or surface proteins. The vaccines are introduced in the body via mouth, injection or by nasal route to incite the immune system against foreign bodies [6].

In the process of immunity development, the body produces antibodies against specific microorganisms, which generates the defense mechanism. When a person encounters the same microorganisms later, the antibodies produced by the body in response to the microorganisms’ antigens either prevents the person from the disease induced by the microorganism or lessens the severity of the disease [6]. Vaccines, in general, are considered the most economical healthcare interventions and its said that “A dollar spent on a childhood vaccination not only helps save a life but greatly reduce spending on future healthcare” [6, 7].

## What are different types of vaccines?

There are different types of vaccines including live attenuated, inactivated, protein-based, nucleic acid, and viral vector-based. Each type of vaccine has a subtle structure, advantages and disadvantages with respect to immunogenicity, safety, ease of use and effectiveness.

### Live attenuated vaccines

“Live attenuated vaccines are viruses weakened by passing through animal or human cells, until genome mutates and is unable to cause disease” [7]. The attenuated virus replicates like a natural infection and causes strong T cell and B cell immune responses [7]. Live attenuated vaccines have the inherent ability to induce toll-like receptors (TLRs) such as TLR 3, TLR 7/8, and TLR 9 of the innate immune system that involves B cells, CD4 and CD8 T cells. They can be obtained from ‘cold adapted’ virus strains, reassortments, and reverse genetics; and can be low-cost and rapidly produced [7]. Herd immunity can be achieved through these vaccines in the community [7]. Broad adjunct testing is required to confirm their safety and efficacy. There is also a possibility of mutations during viral replication which may lead to recombinants post-vaccination [8]. In addition, cold chain distribution in the community is required. Some examples of live attenuated vaccines include BCG, Smallpox, and Polio (OPV) [7]. An example of such vaccine to mitigate COVID-19 is DelNS1-SARS-CoV2-RBD, by University of Hong Kong [7].

### Inactivated vaccines

These are inactivated viruses developed using formaldehyde or heat. They do not have any live component of the viral particles [8]. These are noninfectious, stable, and safer compared to a live attenuated vaccine [8]. These vaccines can be freeze-dried and do not require cold chains for distribution [7]. Such vaccines do not replicate and have a suboptimum immune response. They can be used along with adjuvants to increase their immunogenicity. As large quantities of viruses need to be handled while maintaining their integrity [6–8], there are chances of Th2 cell skewed response (antibody-dependent enhancement, ADE). Some examples of inactivated vaccines include Hepatitis A and Rabies. An example of such a vaccine to mitigate COVID-19 is PiCoVacc, by Sinovac Biotech.

### Protein-based vaccines

#### Protein sub-unit

These are antigenic components [spike (S) protein] generated in vitro. They do not have any live components of the viral particle. They are considered safe and have less adverse effects. They exhibit low immune response, therefore, need multiple dosing and adjuvants. Even memory for future responses is doubtful [6–8]. The S protein of the SARS-CoV-2 is the most suitable antigen to induce the neutralizing antibodies against the pathogen [9]. One of the examples of such a vaccine to mitigate COVID-19 is NVX-CoV2373, by Novavax [7].

#### Virus-like particles

These are empty virus shells without genetic material. They are considered safe, induce a strong immune response, and are difficult to manufacture [7, 8]. One example of such a vaccine to mitigate COVID-19 is Triple-Antigen Vaccine, by Premas Biotech [7].

### Nucleic acid vaccines: new generation vaccines

#### DNA vaccines

These vaccines are made by introducing DNA encoding the antigen from the pathogen into a plasmid (antigenic components of SARS-CoV-2 such as spike protein). These are considered safe, unable to cause disease. These types of vaccines are unproven in practice. They can cause adverse events (ADE) when used alone [6–8]. These vaccines are highly immunogenic; generate a high titer of neutralizing antibodies when given with inactivated vaccine. An electroporation device is needed to deliver these vaccines. One example of such a vaccine to mitigate COVID-19 is INO-4800, by INOVIO Pharma, Korean Institute of Health, and International Vaccine Institute [8].

#### RNA vaccines

RNA vaccines are lipid-coated mRNA of the SARS-CoV-2 expressing spike protein. These are considered safe and unable to cause disease, but are able to induce ADE and are unproven in practice [6, 7]. Examples of such vaccines to mitigate COVID-19 are mRNA-1273, by Moderna; and BNT162 (a1, b1, b2, c2), by BioNTech/Fosun Pharma/Pfizer [8].

### Viral vector vaccines

Recombinant DNA technology is used to create these vaccines. The DNA encoding an antigen from the pathogen is inserted into the bacteria or virus vectors. These bacteria or virus vectors then express the antigen in these cells. The antigens are harvested and then purified from the bacteria or virus vectors. Viral vector vaccines could be replicating or nonreplicating.

#### Replicating

An unrelated virus-like measles or adenovirus is genetically engineered to encode the gene of interest. These are considered safe and are able to induce strong T cell and B cell response. Some examples of such vaccines include Hepatitis B, HPV, and pertussis [6–8].

#### Nonreplicating

An unrelated virus, like measles or adenovirus (with the inactive gene), is genetically engineered to encode the gene of interest. These are considered safe and require booster doses to induce long-term immunity. These types of vaccines are not licensed yet [6, 7]. Examples of such vaccines to mitigate COVID-19 are Ad5-nCoV by CanSino Biological Inc./Beijing Institute of Biotechnology; and ChAdOx-nCoV-19 by the University of Oxford [8].

## How COVID-19 vaccines are developed rapidly as compared to traditional vaccines?

**Fig. 1:**
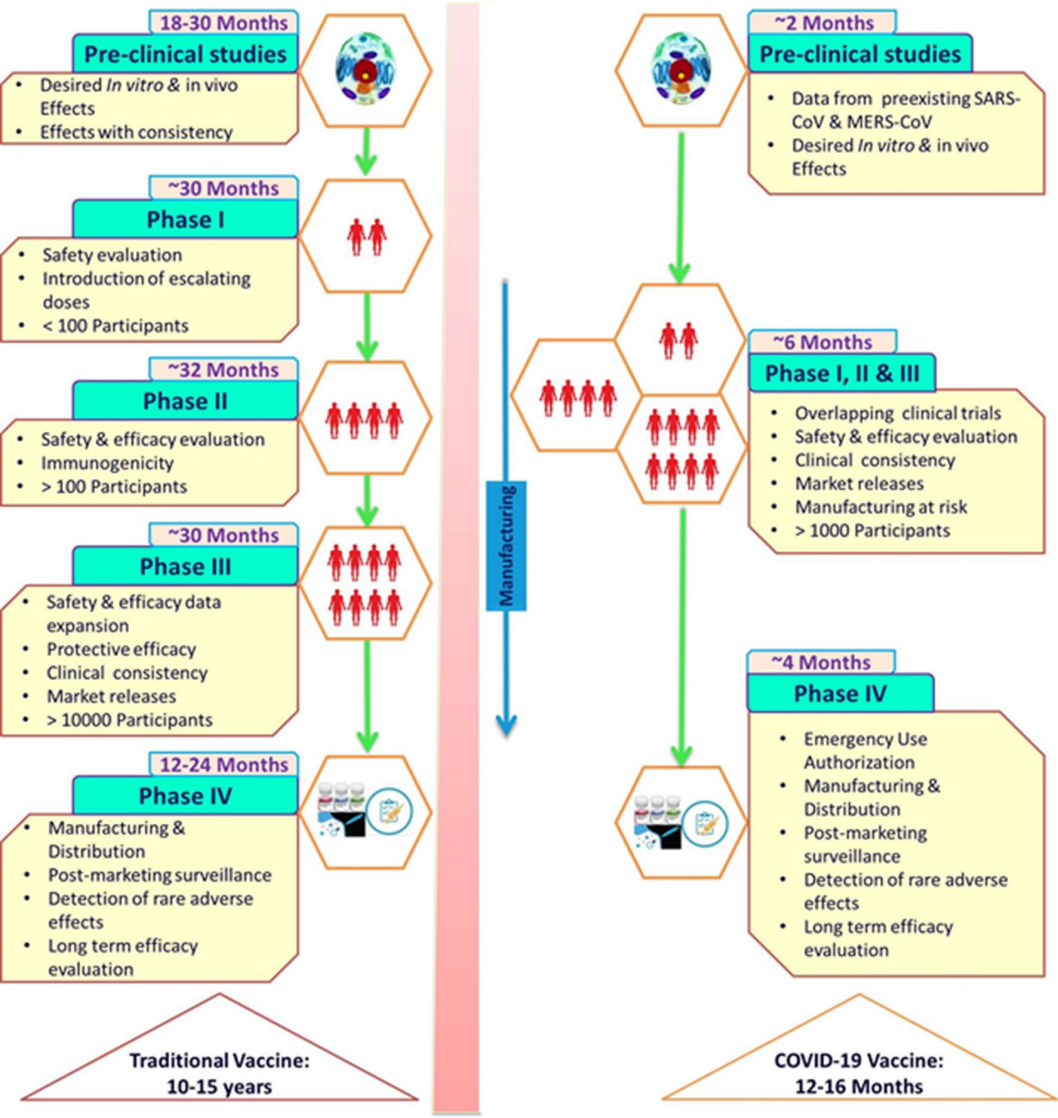
Rapid development of COVID-19 vaccine as compared to traditional vaccine development. Adapted from Calina et al. [76]

Vaccine development is a complex multidisciplinary activity, blending knowledge of host–pathogen interactions with clinical science, population-level epidemiology, and the biomechanical requirements of production. The core is an insight into immune processes that influence the disease and protection and their variation between individuals, risk groups, and populations [10]. Traditional vaccine development (Fig. 1) has been a complex and time-consuming process that typically takes around 10–15 years. Vaccine development usually begins with an exploratory stage focusing on basic research and computational modeling to find out potential natural or synthetic antigens as a vaccine candidate. After this, a pre-clinical study (18–30 months) starts with cell-culture followed by animal studies to analyze the safety and immunogenic potential of the vaccine candidate. After appropriate in vivo results on safety, immunogenicity, and efficacy, human clinical trials initiated for safety and immunogenicity in small groups, and later in the large groups over 3 phases (Phase 1 or I, 2 or II and 3 or III). The primary goal of Phase 1 trial (~ 30 months) is to assess the safety and immunogenicity of the vaccine candidate. In Phase 1 trial, the vaccine is administered to less than a hundred healthy participants. If promising results are obtained in Phase 1, Phase 2 trial (~ 32 months) is carried out in more than a hundred participants, divided into multiple groups by demographics. The goal of the phase 2 trial is to confirm the safety and immunogenicity of vaccine candidates. Also, the suitable dose required for Phase 3 is calculated. If encouraging results in Phase II trials are obtained, Phase 3 trial (~ 30 months) is then carried out in thousands of participants to evaluate the efficacy. “Incidence of disease at the time of phase 3 trials impacts the sample size” [11]. If there is a low incidence of disease in the community, large sample size will be required to satisfactorily decide the vaccine efficacy. After completion of these trials, safety and the clinical efficacy are calculated, then the vaccine is reviewed for approval by regulatory bodies, such as Food and Drug Administration (FDA) of the United States of America (USA), or the European Medicines Agency in European Union (EU). Later, manufacturing and post-marketing surveillance are done after the vaccine is marketed for public use and monitored for general effectiveness within the population. Even after the vaccine is adopted for widespread use, events of adverse effects are recorded. The developer advances the vaccine development only if the data is promising, the risk of failure is relatively low and there is a market for the vaccine [11].

The mumps was the only fastest developed and approved vaccine for use, taking about 5 years. Even with this experience, it is clearly a big challenge to develop a vaccine against COVID-19 in a span of 12–24 months. COVID-19 vaccine development (Fig. 1) has targeted to significantly reduce this 10–15-year timeline to 12–24 months. The initial process started as soon as the genome sequence of SARS-CoV-2 was available. The significant amount of time was saved by using the data from the preclinical development of vaccine candidates for SARS-CoV and MERS-CoV and omitting the initial step of the exploratory phase. Some vaccine candidates used modified production processes from those of existing vaccine candidates while others used preclinical and toxicology data from related vaccines. Therefore, the first clinical trial of CVCs started in March 2020 (NCT04283461) [11]. Clinical trials were designed to reduce the time horizon by overlapping clinical trial phases. The initial phase I/II trials were followed by rapid advancement to phase III trials as soon as the interim analysis of the phase I/II data was completed. The US accelerated the development of five CVCs under the Operation Warp Speed to make them available by the end of 2020 for emergency use and have billions of doses ready by 2021 [11]. Manufacturers prepared themselves to rapidly produce billions of doses and few of them already started the commercial production of vaccines without any results from phase III trials. The review process is expedited via Emergency Use Authorization (EUA) by countries like UK, USA and subsequently followed by many more. The challenging task of developing CVCs is achieved in record time frame of 12–16 months as compared to traditional vaccine development taking 10–15 years (Fig. 1) [12].

## COVID-19 vaccines approved through Emergency Use Authorization

Vaccines traditionally used are live attenuated viruses, inactivated viruses, protein or polysaccharide conjugated subunit vaccines and virus-like particles. Also, recently included vaccines are nucleic acids, DNA and RNA and viral vectors and recombinant proteins.

SARS-CoV-2 induces a strong adaptive immune response of both T and B cell. Additionally, antibodies IgG and IgM appear about 10 days post-infection. The majority of the patients are able to seroconvert in 3 weeks. The antibodies are created against internal nucleoprotein (N) and spike protein (S) of the virion and possess neutralizing activity. Antibodies which bind to the spike protein, particularly to its receptor-binding domain (RBD), inhibit its attachment to the host cell and counteract the virus [13]. Table 1 lists few vaccine candidates approved through EUA that reached up to or completed Phase 3 trials.

### BNT162 vaccine by Pfizer and BioNTech

On December 2, 2020, UK became the first country to approve COVID-19 vaccine BNT162 developed by Pfizer and BioNTech via EUA. On December 11, 2020 US FDA issued first EUA for BNT162 having demonstrated 95% efficacy in preventing disease in phase III clinical trial results [14]. Later Canada and Mexico also approved BNT162 via respective EUA pathways. On December 31, 2020, WHO approved first vaccine candidate, BNT162, for emergency use thereby making it easier to manufacture and distribute this vaccine globally [15]. Initially, four candidates were developed of which two were nucleoside modified mRNA, modRNA; one was uridine containing mRNA, uRNA; and other was self-amplifying mRNA, saRNA. In the preclinical study, modRNA BNT162b2 showed protective antiviral effects in *Rhesus macaques* with concurrent elevated neutralizing antibody titers and a Th-1 biased cellular response in *Rhesus macaques*, as well as in mice. Therefore, BNT162b2 was selected for Phase 2/3 clinical trials [16].

In Phase 1/2 trial of two hundred participants aged 18–55 years with a vaccine dose range of 1–100 µg is currently recruiting (NCT04380701) as is a Phase 2/3 trial of about 32,000 participants (NCT04368728) and a Phase 1/2 trial of 160 participants between age 20–85 (NCT04588480) [16].On November 9, 2020, Pfizer and BioNTech declared interim results of 94 participants of Phase 3 trial claiming > 90% efficacy of BNT162b2 against SARS-Cov-2 infection at 7 days after the administration of second dose [16]. Phase 1 trial data showed similar immunogenicity between BNT162b1 and BNT162b2, while BNT162b2 was associated with a lower incidence and severity of systemic reactions than BNT162b1 [17].

Another study of Phase 1/2 data for BNT162b1 (NCT04368728) showed robust immunogenicity at all three doses of 10 µg, 30 µg and 100 µg among 45 participants, 18–55 years of age. Adverse reactions were high at the maximum dose and therefore, participants were not given a second dose. Participants who were given two doses between 1 and 50 µg of BNT162b1 had vigorous receptor-binding domain (RBD)-specific IgG antibody, T-cell and favorable cytokine responses [18].

Both BNT162b1 and BNT162b2 received the FDA Fast Track designation. But BNT162b2 was preferred over BNT162b1 for Phase 2/3 safety study, based on preclinical and clinical study results. The developers have asked the FDA to consider an expanded protocol for the Phase 3 trial to include up to 44,000 participants. Europeans Medicines Agency (EMA) has initiated a rolling review of BNT162b2 which helped to accelerate its approval [16]. One drawback with this vaccine is that it requires storage at − 80° to − 60 °C, a fact that could pose logistic problems [19].

### mRNA-1273 vaccine by Moderna

Moderna’s mRNA-1273 becomes the second CVC to be approved by FDA under EUA. It is developed on the basis of available data of coronaviruses causing severe acute respiratory syndrome (SARS) and the Middle East respiratory syndrome (MERS). A Phase 3 trial of 30,000 participants at higher risk for COVID-19 is ongoing. Participants will be given 100 µg dose of mRNA-1273 or placebo and then be followed for up to 24 months (COVE trial; NCT04470427). After successful completion of Phase 1 trial (NCT04283461) of 105 participants, Phase 2 trial of 600 participants evaluating 25 µg, 100 µg and 250 µg dose levels of the vaccine was carried out (NCT04405076). Then, Phase 3 results of 95 participants after an interim analysis revealed 94.5% efficiency of the vaccine with no significant safety concerns [20].

The mRNA-1273 effectively produced neutralizing antibody titers in 8 participants of Phase 1 trial after receiving 25 µg or 100 µg doses. Neutralizing antibody titers of these participants were similar to the convalescent sera from COVID-19 recuperated patients [21]. Higher age adults subjects who received two doses of either 25 µg or 100 µg of the mRNA-1273 demonstrated safety and suffered mild or moderate effects including, fatigue, chills, headache, myalgia, and pain at the injection site [22]. In a preclinical study, mRNA-1273 prevented viral replication in the lungs and produced neutralized titers similar to subjects receiving 25 µg or 100 µg doses of the vaccine [23]. Another preclinical study consisting of nonhuman primates challenged with SARS-CoV-2 showed neutralizing activity and reduced inflammation and lung activity post administration of mRNA-1273 [24].

The mRNA-1273 also got the Fast Track designation from the US FDA. A Phase 3 trial of the vaccine is currently underway and is funded by the Operation Warp Speed [25]. One potential issue for this vaccine could be the storage temperature requirement of − 25° to − 15 °C is required [19].

### AZD1222 by AstraZeneca and University of Oxford

On December 30, 2020, UK and on January 2, 2021, India approved AZD1222 COVID-19 vaccine developed by AstraZeneca and the Oxford Vaccine Group at the University of Oxford. It was previously called as ChAdOx1, a chimpanzee adenovirus vaccine [26, 27]. This group has previously developed a MERS vaccine. In India, this vaccine is jointly developed by Serum Institute of India and AstraZeneca and is branded as Covishield. A preclinical study showed significantly reduced viral load and humoral and cellular immune response [28]. Another preclinical study demonstrated an immune response in both mice and pigs [29]. ChAdOx1, a replication-deficient simian adenoviral vector expressing the full-length SARS-CoV-2 spike (S) protein, was commenced in April 2020 following preclinical studies involving non-human primates using a single dose. When one vs two doses of ChAdOx1 in both mice and pigs were compared, a single dose induced antigen-specific antibody and T cells responses, and a second booster dose enhanced antibody responses, particularly in pigs, with a significant increase in the level of SARS-CoV-2 neutralizing titers [29].

A Phase 1/2 (NCT04324606) study involving 1077 healthy adult participants aged 18–55 years, assessed the safety, reactogenicity, and immunogenicity of a viral vectored coronavirus vaccine, expressing the spike protein of SARS-CoV-2. The results demonstrated an acceptable safety profile for ChAdOx1 nCoV19 and increased antibody response by homologous boosting [30]. A Phase 3 trial (NCT04516746) is ongoing and has enrolled more than 40,000 subjects. Preliminary results have demonstrated that the safety profile of the vaccine candidate is acceptable, with most patients demonstrating an antibody response after one dose and all patients showing a response after two doses [30]. A Phase 3 trial in Brazil reported one death, which was confirmed by the Brazilian National Health Surveillance Agency (ANVISA). AstraZeneca stated that the results from the Phase 3 trial demonstrate immunogenicity, but have not yet publicly released any data [31]. An inhaled version of the vaccine candidate is also being tested in a small trial involving 30 participants [31].

The trials by AstraZeneca are funded by BARDA and Operation Warp Speed. IQVIA also announced they are partnering with AstraZeneca to advance clinical trials for this vaccine. Phase 3 trials are being conducted in the United States and India but were put on hold following reporting of a serious adverse event. Trials have since restarted. Additionally, EMAs human medicines committee (CHMP) and Health Canada have initiated a rolling review of AZD1222 to reduce the decision-making time related to safety and efficacy. The Australian Therapeutic Good Administration (TGA) granted AZD1222 provisional determination, the first step in the approval process. In Britain, the Medicines and Healthcare products Regulatory Agency (MHRA) has also started an accelerated review of AZD1222 [31]. This vaccine requires refrigeration (2–8 °C), which can potentially be problematic for use in low-income countries [19].

### CoronaVac by Sinovac

CoronaVac (formerly PiCoVacc) is approved by China through EUA. CoronaVac is a formalin-inactivated and alum adjuvanted vaccine candidate developed by Sinovac Biotech, China [32]. Results from preclinical studies showed partial or complete protection in non-human primates exposed to SARS-CoV-2 [33].

A Phase 1/2 trial of 743 healthy participants (18–59 years old) who received two different dosages of the vaccine or placebo is active but not recruiting (NCT04551547). A Phase 1 trial of 143 participants (NCT04352608) and Phase 2 trial of 600 participants (NCT04383574) are both active but not recruiting. Phase 3 trial is underway (NCT04456595) to have 9000 participants. Trials are also ongoing in Turkey (NCT04582344) and in Indonesia (NCT04508075). Phase 1/2 trials revealed that the vaccine has good safety and immunogenicity with seroconversion occurring in 92.4% of participants after the 3 µg dose given on a 0–14 day schedule and 97.4% of participants with the same dose on a 0–28 day interval [34].

Preliminary results from the Instituto Butantan trial, declared by the Sinovac, showed CoronaVac is safe with no reported serious adverse events. However, the trial in Brazil was briefly suspended due to patient death, though the trial has resumed later [35].

### COVID-19 vaccine by Sinopharm and the Wuhan Institute of Virology, China

China approved this vaccine via EUA. Sinopharm and Wuhan Institute of Virology under the Chinese Academy of Sciences have developed an inactivated CVC [36]. A Phase 1/2 clinical trial (ChiCTR2000031809) involving healthy subjects is ongoing. According to a release from China National Biotec Group, this vaccine has demonstrated a strong neutralizing antibody response. Phase 1 and Phase 2 trials data also showed immunogenicity [37]. A Phase 3 trial is in progress in Peru, Morocco, and United Arab Emirates.

### Sputnik V by the Gamaleya Research Institute, Russia

Russia has approved first CVC as Sputnik V (previously as Gam-COVID-Vac). The Gamaleya Research Institute in Russia and Health Ministry of the Russian Federation are assessing their non-replicating viral vector vaccine, Sputnik V, in a Phase 3 trial. However, there is no trial data available to date. This led to criticism as even there is a lack of data on safety and efficacy, the vaccine is approved.

Two Phase 1/2 trials with 38 subjects each were conducted (NCT04436471, NCT04437875). Sputnik V is additionally being evaluated in a small Phase 2 trial with 110 subjects older than 60 years (NCT04587219). A Phase 3 trial with about 40,000 participants is also in progress (NCT04530396). Aside from Russia, Sputnik V is also being evaluated in Belarus (NCT04564716) and the United Arab Emirates. The results from the Phase 1/2 trials demonstrated the safety and immunogenicity of the vaccine [38]. The Russian Direct Investment Fund also announced that Sputnik V is 92% effective based on the interim trial results from 20 participants. A preliminary pre-submission of the vaccine has also been proposed in Brazil [39].

### BBIBP-CorV by Sinopharm and Beijing Institute of Biological Products, China

BBIBP-CorV is inactivated CVC developed by Sinopharm in association with Beijing Institute of Biological Products, China. Firstly China and later on United Arab Emirates (UAE) approved the vaccine through EUA [36].

BBIBP-CorV is currently being assessed in Phase 2 (CHiCTR2000032459) and Phase 3 trial in China (ChiCTR2000034780) as well as Phase 3 trial in Argentina (NCT04560881). BBIBP-CorV is shown to be highly effective in preventing disease against SARS-CoV-2 in *Rhesus macaques* [40]. Phase 1 results showed that BBIBP-CorV was safe and tolerated at all dose levels, with all participants showing a humoral response to the vaccine after 42 days. The UAE announced that the vaccine is 86% effective [41].

### EpiVacCorona by Federal Budgetary Research Institution State Research Center of Virology and Biotechnology, Russia

Russia also granted regulatory approval to EpiVacCorona, a peptide vaccine candidate for COVID-19, developed by Federal Budgetary Research Institution State Research Center of Virology and Biotechnology [42].

A Phase 1/2 trial in Russia is assessing the effectiveness of the vaccine (NCT04527575). A Phase II clinical trial of the vaccine was completed recently. Head of the zoonotic diseases and flu department with the State Research Center of Virology and Biotechnology has said “participants have developed immunity a month after the first vaccination” [43], but there is no data available in the public domain.

### Covaxin by Bharat Biotech and National Institute of Virology, India

On January 2, 2021, India approved an inactivated vaccine called Covaxin, developed by Bharat Biotech and India’s National Institute of Virology [27]. A Phase 1/2 trial of about 1100 healthy subjects is ongoing after obtaining permission from the Drug Controller General of India. The Indian Council of Medical Research (ICMR) reported that Covaxin has entered Phase 2 clinical trials. On October 27, 2020, the ICMR approved Covaxin for Phase 3 trial. Results of a two-dose regimen study administered to *Rhesus macaques* demonstrated an increase in SARS-CoV-2 specific IgG and neutralizing antibodies as well as diminished viral replication in the nasal cavity, throat, and lungs [44]. According to the trial principal investigator, initial results from the first fifty participants who received the vaccine seem to be promising. In addition, according to Bharat Biotech, the first two phases of the trial did not demonstrate any major adverse events [45]. The proposed distribution for this vaccine is February 2021, according to an ICMR scientist who spoke with Reuters.

## COVID-19 vaccines under clinical trials

Some of the potential CVCs that are in Phase 3 clinical trials and might get EUA approval are described below. The other CVCs that are in clinical trials are listed in Table 2.

### JNJ-78436735 by Johnson & Johnson

Johnson & Johnson (J&J) is developing JNJ-78436735 (Previously as Ad26.COV2.S), using their AdVac and PERC6 systems, also used to develop the Ebola vaccine. In partnership with BARDA, J&J has promised to invest more than $1 billion in vaccine research and development. JNJ-78436735 is currently funded by Janssen, BARD, NAID and the Operation Warp Speed [46].

A randomized, double blind, placebo-controlled, Phase 1/2 study of recombinant JNJ-78436735 in 1045 healthy subjects, 18–55 years of age, and in adults 65 years or older is ongoing. Study sites are selected in the US and Belgium (NCT04436276). The Phase 3 ENSEMBLE trial will enroll up to 60,000 subjects in the US and other countries (NCT04505722). The study protocol for the Phase 3 ENSEMBLE trial was released by J&J on September 23, 2020. Results from the Phase 1/2 study showed that a single dose of the vaccine was safe and immunogenic [47]. The results of the preclinical study showed that a single injection of JNJ-78436735 produced a strong neutralizing antibody response and offered complete or near-complete protection in bronchoalveolar lavage and nasal swabs after SARS-CoV-2 administration in *Rhesus macaques* [48]. Another preclinical study in hamsters indicated that the vaccine protected against severe disease when tested [49].

On June 10, 2020, J&J announced it is fast-tracking the Phase 1/2 trials. The ENSEMBLE trial was on hold pending a review of an adverse event, but J&J has been cleared to resume the trial in the US and Brazil after clearance from the Independent Data Safety and Monitoring Board. J&J also plan to start testing its vaccine in adolescents as soon as possible [46]. This vaccine candidate requires storage at 2–8 °C [19].

### Ad5-nCoV by CanSino Biologics

China’s CanSino Biologics has developed a recombinant novel coronavirus vaccine that incorporates the adenovirus type 5 vector (Ad5) called Ad5-nCoV. A Phase 1 clinical trial in China involving 108 participants, 18–60 years old, is active, but not recruiting. In this trial the participants will receive low, medium, and high doses of Ad5-nCoV (NCT04313127). A Phase 1 trial in China is also assessing intramuscular as well as mucosal vaccination of Ad5-nCoV across two doses (NCT04552366).

A Phase 1/2 trial involving 696 participants in Canada is registered and not yet recruiting (NCT04398147). A Phase 2 double-blind, placebo-controlled trial with 508 participants in China (NCT04341389) is active but not recruiting. A phase 2b trial in China is evaluating the safety and immunogenicity of Ad5-nCoV in participants who are 6 years of age and older (NCT04566770). A Phase 3 trial in Russia with 500 participants across multiple study centers is ongoing (NCT04540419). A Phase 3 trial involving 40,000 participants in countries including Pakistan, Saudi Arabia and Mexico is also ongoing (NCT04526990). A single dose of Ad5-nCoV vaccine protects against upper respiratory infection of SARSCoV-2 in ferrets. Results from the Phase 1 trial showed a humoral and immunogenic response to the vaccine. Adverse reactions such as pain (54%), fever (46%), fatigue (44%), headache (39%), and muscle pain (17%) were reported in 83% of the patients in the low and medium dose groups and in 75% of the patients in the high dose group. Results from the Phase 2 trial showed neutralizing antibodies and specific interferon γ enzyme-linked immunosorbent assay, at all dose levels for most of the participants. On June 25, 2020, China’s Central Military Commission announced that Ad5-nCoV can be used in the military for a period of 1 year.

### NVX-CoV2373 by Novavax

In March 2020, Novavax announced that it has manufactured a stable, prefusion protein nanoparticle vaccine candidate for COVID-19. A Phase 1/2 trial evaluating NVX-CoV2373 commenced on May 25, 2020 [50].

A randomized, observer-blinded, placebo-controlled trial involving 130 healthy participants, 18–59 years of age, is ongoing at two sites in Australia. In this trial, patients will receive a two-dose regimen of 5 µg or 25 µg of NVX-CoV2373 with or without Novavax’s Matrix-M adjuvant (NCT04368988). A Phase 2b trial is also ongoing in South Africa, with two cohorts, group of 2,665 healthy adults and group of 240 HIV positive adults (NCT04533399). Phase 1 trial participants who received the vaccine developed an antibody response at multiple doses. NVX-CoV2373 was also reported to be safe [51].

Novavax received the Fast Track Designation from the FDA for NVX-CoV2373 [52]. On May 11, 2020, CEPI announced that they had provided Novavax with $384 million for the development and manufacturing of NVX-CoV2373. Novavax plans to produce 1 billion doses of NVX-CoV2373 by 2021 as part of their latest acquisition of Praha Vaccines. Novavax was also awarded a $60 million US Department of Defense contract towards manufacturing NVX-CoV2373, and another $1.6 billion from Operation Warp Speed, if the candidate will be proved effective in clinical trials [53]. A Phase 3 trial has also begun in the United Kingdom, which will evaluate the vaccine in 10,000 participants. Novavax provided an update on October 27, 2020, of its Phase 3 trial of NVX-CoV2373 in North America, stating that the trial would commence at the end of November, roughly one month later than expected [53].

## COVID-19 vaccines: challenges and future prospects

### Ethics

The vaccine development effort over the globe for the COVID-19 pandemic is unprecedented, in terms of scale, speed, and supply chain. It is made possible to have a safe and effective vaccine available by the end of the year 2020, for the more vulnerable group of the population and hopefully in the first half of 2021 to all the others. Operation Warp Speed program was introduced in US to fast-track vaccine development. Moderna’s mRNA vaccine and AstraZeneca/University of Oxford’s AZD1222 vaccine are part of this program. Classical clinical efficacy trials of vaccines usually enroll thousands or tens of thousands of healthy participants. However, to accelerate the COVID-19 vaccine development, clinical trial phases were combined, and smaller population was enrolled. This is a noteworthy concern when the vaccine is supposed to be given to people throughout the world, there could be emergence of unknown side-effects in the larger population, which were previously not witnessed in smaller groups during short-term trials. It is important to consider whether there was an appropriate demographic consideration in the design of the clinical trials including, different races, varying age groups and those with comorbidities, as the exclusion of these may lead to unforeseen outcomes upon vaccinating these individuals when the vaccine is released for public use.

The production teams of the vaccine candidates have stated to be under pressure to develop a vaccine within few months as compared to the conventional process of 10–15 years. With a fast-track process, post-marketing surveillance turns out to be important. Post-marketing surveillance would ensure that the vaccines are observed for side effects when administered in diverse populations. The foremost ethical concern is to find a safe and effective vaccine but at the same time not exposing clinical trial participants to avoidable risks [54].

Fast-tracking of vaccines may turn unfavorable as it could result in ineffective vaccine and may only provide partial or no immunity to some vaccinated persons. Although it is assumed that there will be thorough inspection of the vaccine candidates for safety and efficacy from the scientific community before vaccine is released for administration into the public. It is important to consider the recent small trials of the Russian vaccine Sputnik V as well as the Chinese vaccine candidates. Both Russia and China have begun the mass rollout of state-sponsored vaccine candidates with limited data. In the perspective of a public health emergency of international concerns, such shortened regulatory pathways and fast-tracked implementations are still commonly regarded as experimental interventions and are unique. However, to preserve public trust in vaccines, it is vital that complete transparency in all facets of vaccine development is available.

Due to increased demand and limited supply of vaccines, several countries including the US, India, and Europe have decided that the vaccines will be provided first to their own citizens. However, questions are being raised concerning the ethics associated with fair allocation. Though AstraZeneca has announced a collaboration with Serum Institute of India to provide an adequate number of doses to low and middle-income countries, it will be interesting how the allocation will be done when the vaccine candidates are approved and becomes available. It is also crucial to prioritize certain groups of people for vaccine allotment including, health care workers, immunocompromised individuals, those with comorbidities, the elderly, and those with lower socioeconomic status to guarantee distributive justice. There are also worries that the political pressure to hasten the development and approval processes, may result in an ineffective vaccine being released to the public. Such a consequence may lead to the public being hesitant from receiving future vaccines [55].

To date, no trials for COVID-19 vaccine has focused on pregnant women, despite being deemed a vulnerable population by the US Centers for Disease Control and Prevention (CDC). Although there are unanswered questions regarding the safety and efficacy of COVID-19 vaccines in pregnant women, FDA-approved COVID-19 vaccines should not be refused to women solely based on their pregnancy or lactation status, when they otherwise meet the conditions for vaccination. Patient-provider discussions should also consider the patient’s individual risk–benefit profile concerning exposure at work or at home, risk to expose other members of their household, current health status and perceived risk of COVID-19 associated impediments [56]. Pregnant women should get COVID-19 vaccine without delay, as the consequences of COVID-19 infections in pregnancy are equivalent or worse than in non-pregnant populations. There is potential for damage to not one but two lives, and females of childbearing potential may have heightened workplace exposure to SARS-CoV-2. Additionally, the ongoing vaccine trials should include pregnant women to test vaccine candidates’ study safety and efficacy [57].

### Vaccine efficacy

Vaccine effectiveness is described as the protection provided by immunization in a defined population. It includes both direct (vaccine-induced) and indirect (population-related) protection. The effectiveness of a vaccine is proportional to its efficacy but is also influenced by the vaccine coverage, access to healthcare centers, associated costs, and other factors not directly related to the vaccine [58]. The question is, how much efficacy is actually needed for a vaccine to be considered immunogenic? Though more research is required, preliminary research studies have revealed that efficacy of > 70% is desired to eradicate the infection. A preventative vaccine with an efficacy of < 70% will still have a major effect and may add to obliterating the virus, given proper social distancing measures. Vaccines with an efficacy below 70% may contribute to decreasing the length of infection. Another study with simulation experiments showed that to prevent a pandemic, the vaccine efficacy has to be at least 60% with 100% vaccination coverage. The vaccine efficacy threshold rises to 70% when coverage drops to 75% [59].

Phase III clinical trials are required for all vaccine candidates to demonstrate that they are effective and safe in a larger population. In addition, the majority of vaccine candidates currently in clinical trials are administered intramuscularly. Though this administration route induces a strong IgG response, which is believed to protect the lower respiratory tract, unlike natural infection, it does not initiate the secretory IgA responses required to protect the upper respiratory tract [11]. Thus, most vaccines will provide protection against infection of the lower respiratory tract and not induce sterilizing immunity in the upper respiratory tract. This could lead to protection from symptomatic diseases but might still allow virus spread by infected person. Thus, a vaccine that could induce sterilizing immunity in the upper respiratory tract would be preferable to stop virus spread. Live attenuated vaccines or viral vectors that can be administered intranasally, would probably also lead to a strong mucosal immune response as well as an IgG response. Alas, very few vaccines that are appropriate for intranasal administration are undergoing development and none have made it to the clinical trials yet [11].

The next ethical question is, what will be the effect of the vaccine on older individuals who are at higher risk from COVID-19? According to Sinovac’s inactivated vaccine and Pfizer’s mRNA vaccine, the effect of the vaccine in older individuals is less compared to younger adults. Thus, there is a need for different vaccine formulation or a booster dose to improve immune responses in older individuals [11]. The children usually show increased reactogenicity compared to adults. As many CVCs have fairly strong adverse effects, low-dose vaccines might be required for children, particularly for AdV and mRNA-based vaccines. Pfizer has considered this approach and accordingly reduced the reactogenicity of its mRNA vaccine in older adults, making it appropriate for children [11].

There is also risk of vaccine enhanced disease for inactivated vaccine candidates (VAERD) that need to be considered. The higher numbers of antibodies are unable to neutralize the virus in case of high viral load, resulting in VAERD. Furthermore, ADE has been observed with other coronaviruses including MERS-CoV and SARS-CoV and could be a risk for CVCs. ADE occurs when antibodies bind to the virus and the resulting antibody-virus complex facilitates viral entry by host macrophages instead of neutralizing the virus. However, when there is an urgent need for CVCs globally, being concerned and assessing such risks should not prevent the release of otherwise safe and effective vaccines to the public [60].

If there is an incidence of the adverse reaction, there should be programs in place to safeguard proper medical treatment and compensation is provided to affected individuals and records are kept for re-evaluating the safety of the vaccine(s). The accountable authorities should also ensure that an effective and fair policy is in place, for instances where vaccination is compulsory, so the public trust in the health care system is not risked. Pre-existing immunity to adenoviruses is a concern, specifically for those vaccine candidates utilizing human adenoviruses such as CanSino’Ad5 vaccine, as it may lead to a decreased immune response to the vaccine. AstraZeneca/Oxford’s AZD is another adenovirus-based vaccine candidate, but instead of utilizing adenovirus derived from humans, it utilizes a genetically modified chimpanzee-derived adenovirus. This effectively eliminates the concern about pre-existing immunity and thus, averts the negative impact on the immune response generated to the vaccine [60]. Although some vaccines are approved through EUA, long-term data on vaccine safety is also crucial. The well-known case of Dengue vaccine should not be overlooked, where dengue vaccine protected individuals against virologically confirmed dengue (VCD) and severe VCD for 5 years, who had exposure to dengue prior to vaccination. There was also a higher risk of VCD and severe VCD in vaccinated individuals who were not exposed to dengue earlier [61]. Thus, to avert such obstacles after vaccination, even after EUA approval, long-term safety and efficacy data is essential.

Furthermore, if a vaccine is approved for use but subsequently it is found to be not as effective as expected in the population, it could lead to a loss of trust in the vaccines. There are reports of few adverse effects with the Pfizer vaccine (Table 3) [62–64] and these recent adverse reactions were confirmed by the Finnish Medicines Agency Filmea, Finland [65]. Thus, when an effective vaccine is launched, fewer people may be inclined to accept it, which in turn can lead to further worsening of the pandemic and a decline in the confidence in already approved and effective vaccines against infections. Hence, it is vital to building trust in the public health system by being completely transparent and reporting accurate data in a timely fashion [61]. Thus, the ideal characteristics of CVCs described by WHO are important to consider while developing vaccines (Table 3) [66, 67].

**Table 3:** Few mild side effects of Pfizer/BioNTech COVID-19 vaccine that should not last more than a week [60–62] and Ideal COVID-19 vaccine characteristics according to WHO [64, 65]

Few mild side effects of (Pfizer/BioNTech) COVID-19 vaccine	Ideal COVID-19 vaccine characteristics according to WHO
Injection Site pain	An admirable safety of vaccines throughout target population No contraindications
Injection Site swelling	Least adverse incidents that are weak and temporary
Injection Site redness	Be appropriate for administrations to all target population
A headache	Generate protective immunity- preferably after one shot
Fever	Produce protective immunity quickly after 14 days
Chills	Vaccine with no less than 70% efficacy
Tiredness	Not elicit immunopathology or evidence of antibody-enhanced disease (ADE)
Muscle pain	Generate protection in high risk profile peoples Deliver long term protection with both humoral and cell-mediated immunity for no less than 12 months
Joint pain	Booster dose requirement no less than 12 months
Nausea	Be rapidly produced at cost or dose that permits wide-ranging use
Swollen lymph nodes (lymphadenopathy)	Be thermostable, to be stored at room temperature to enhance vaccine distribution and availability
Remote chance of Severe allergic reaction	Be administered through non-parenteral mechanisms for ease and other logistical issues
	Be co-administered with other vaccines

## Manufacturing and distribution

Manufacturers have a valuable share in the vaccine supply chain as their credibility rest on the effectiveness of their vaccines. The risks of poorly performing supply chains are detrimental for the safety and effectiveness of the vaccines, with potential consequences for future supply in case of adverse events [68]. Manufacturers from developing countries are disparate in nature and are either privately or state-owned [68]. To ensure that the threat of COVID-19 is eliminated, it is critical that a coordinated and cooperative approach is taken. This includes collaboration between several international organizations to safeguard sufficient financing and fair distribution of the vaccine supply. The organizations such as Developing Countries Vaccine Manufacturers Network (DCVMN), The Global Alliance for Vaccines and Immunizations (GAVI), Global Vaccine Action Plan (GVAP), Coalition for Epidemic Preparedness Innovations (CEPI), COVID-19 Vaccine Global Access Facility (COVAX), Bill and Melinda Gates Foundation and WHO are working in tandem to overcome this epidemic. DCVMN is a public-health-driven alliance that represents vaccine manufacturers from developing countries engaged in research, development, manufacturing and vaccine supply for domestic and international use. They aim to protect all people against known and emerging infectious diseases [69]. The number of vaccines supplied collectively by DCVMN members in 2018–2019 was about 3.5billion doses. DCVMN is working in partnership with global health authorities, international organizations and vaccine developers to support the advancement of COVID-19 vaccines. This will allow to rapidly manufacture, fill-finish and supply needed COVID-19 vaccines. Nonetheless, details about the capability for quality control, supply chain and delivery abilities must to be closely assessed [69]. To progress the supply chain, an expert group of representatives of DCVMN prioritized three main areas as Traceability in the context of global digital health initiatives, amassing in the context of addressing vaccine shortages, stock-outs, outbreaks and epidemic prevention, and new packaging technologies. It is imperative that vaccine manufacturers are actively involved in worldwide stakeholders forums as equal partners in determining the best practices for improving the vaccine supply chain [68].

The GAVI is a global public–private partnership to ensure that individuals from emerging countries, mainly children, have access to immunizations. GAVI is also a part of the recent Global Vaccine summit, which allocated funding for COVID-19 vaccine development along with to healthcare systems of GAVI eligible countries to ensure sufficient supply for emerging countries [70]. GVAP unanimously supported by the World Health Assembly in 2012, outlined a bold strategy to improve immunization. It created a Monitoring and Evaluation/Accountability (M&E/A) to track and drive growth. Nevertheless, there is noteworthy improvement to upsurge the visibility for immunization and the benefits of the GVAP M&E/A framework. Only few limitations are needed to be circumvented such as the limited ownership by countries and other stakeholders leading to inadequate implementation of the strategy and poor culpability for achieving GVAP targets. It could hasten the immunization cover in pandemic situations like COVID-19 [71].

Bill and Melinda Gates Foundation have allocated $250 million towards vaccines development and for supporting the health care systems of Sub-Saharan Africa and other emerging countries. CEPI is a foundation involved in financing vaccine development and has launched COVAX in order to allow for equal accessibility of the COVID-19 vaccine for all countries. WHO is also involved in all aspects of thwarting the COVID-19 pandemic. WHO is also recording data from vaccine candidates in its Draft Landscape of COVID-19 vaccine and periodically updates it. Additionally, cooperation from individual countries is equally crucial in the fight against COVID-19 [55].

In the past, platforms based on nucleic acids such as DNA and RNA have not resulted in a successful vaccine for human diseases and so, it is yet to be seen how mRNA vaccines that are temperature-sensitive may pose difficulties for scaling up production. Moreover, for DNA vaccines, its dependence on electroporation or an injector delivery device for vaccine administration is a probable concern. Although, electroporation is considered to be a safe procedure and is vital to generate an enhanced immune response, it can complicate the vaccine delivery [60]. The global vaccine Summit has also called for an equal allocation of vaccines whenever a vaccine is released. There is still a concern that some countries will want to secure the vaccine supply for their citizens first. An example of this is the recent stockpiling of the drug, Remdesivir, in the US. This drug is used for the treatment of patients infected with COVID-19 [60]. Swift large-scale manufacturing of vaccines still remains a challenge with loads of ambiguity to meet the demand. It is likely that two doses of vaccine will be necessary. In this case, at least a 16 billion doses will be needed to meet the worldwide demand. Various vaccines described in this article are being developed by entities that have never manufactured a vaccine. Therefore, unanticipated problems with scaling could cause setbacks. It is also not yet clear whether bottlenecks will occur in the availability of supplies including, syringes or glass vials; how vaccines will be distributed worldwide; and how rollout will occur within different countries [11].

WHO has developed the Emergency Use Assessment and Listing Procedure (EUAL) to accelerate the accessibility of vaccines required in public health emergency situations. It will monitor UN procurement agencies and Member States on the suitability for use of a particular vaccine in the framework of public health emergency, based on minimum available quality, safety and efficacy data. It will speed up the acceptance and rollout of these vaccines in member countries, specifically in low and middle income countries [72, 73]. Vaccine immunogenicity and efficacy is dependent upon how they are packaged, stored, prepared and administered. Vaccines must be kept in the proper cold chain; the cold chain must be appropriately examined; and vaccines must be used only within critical time points after removal from the cold chain or once a multi-dose vial is punctured [19].

### Vaccine hesitancy

Vaccine hesitancy is defined as a delay in acceptance or denial of vaccination regardless of the accessibility of vaccination services. Vaccine hesitancy is complicated and context-specific, differing across time, place, and vaccine to vaccine. It is affected by factors such as complacency, convenience, and confidence [74]*.* If there is greater hesitancy, it can lead to reduced vaccine demand. However, low levels of hesitancy do not certainly mean a higher vaccine demand. The vaccine hesitancy determinants matrix illustrates the factors affecting the behavioral decision to accept, delay or reject some or all vaccines, beneath three categories namely contextual, individual and group, and vaccine/vaccination-specific influences [74].

Protective behaviors are critical to controlling epidemics, and vaccines could be the key for COVID-19. If a COVID-19 vaccine comes to be available, it will be a key public health strategy to reduce the overall COVID-19 burden [75]. However, the anti-vaxxers community always poses a threat and is already countering the statements by experts related to the vaccines. Misleading beliefs of anti-vaxxers and their effects overlaid the path for the nastiest measles eruption in the US in 2019. Now many peoples fear similar outcomes for COVID-19. One poll in US in May 2020, demonstrated that 14–23% of the Americans are not willing to be vaccinated, whereas another poll showed that only 49% of the Americans are willing to take the COVID-19 vaccine. Yet, another study from June 2020, showed nearly 70% of the adults in the US would be willing to take COVID-19 vaccine, if one becomes available. Other countries like Germany and Australia too have a fair share of anti-vaxxers. Hence, there should be a strategy to improve the vaccine acceptance rate in public and to counteract vaccine hesitancy [75]. Frontline healthcare workers play a decisive role in ensuring that all age groups get the recommended immunizations, and by educating people about the importance of immunization [58]. For example, the support for mandatory influenza vaccination in Denmark was significantly less [76]. The reasons for lack of vaccine uptake included considerations by employees that they do not get sick often, the vaccine was not regarded as essential, forgetfulness, and/or lack of time. Only 37.8% were in favor of mandatory influenza vaccination [76]. Thus, educational campaigns regarding benefits offered by vaccines can be helpful.

An online survey of 566 Individuals from Chile to assess an individual’s willingness-to-pay (WTP) for a hypothetical COVID-19 vaccine utilized a contingent valuation methodology. The factors that positively influenced the WTP included pre-existing chronic diseases, knowledge of COVID-19, sickness associated with COVID-19, perception of government performance, income and employment status. The factors that negatively influenced the WTP included belonging to a private health system, not adjusting to work from home with children due to quarantine, and recovery from COVID-19 associated infections. In addition, there would be costs associated with manufacturing and distribution, and the developing laboratories should be financially compensated. Thus, the WTP results from this study can serve as an incentive model for the vaccine developers [3].

The impact of immunization is measured by directly assessing the effects on the vaccinated individual, indirectly on the unvaccinated community—whether herd protection is achieved or not, the epidemiology of the pathogen like altering circulating serotypes or prevention of epidemic cycles, and the added benefits rising from the better health. Aside from the protection of the individual, the larger success of immunization is dependent on attaining a level of coverage enough to interrupt microbial (virus, bacteria, etc.) transmission. Diminished coverage is certainly linked to the resurgence in disease, with outbreaks possibly leading to substantial morbidity and loss of life. The sustained success of immunization programs is the responsibility of all involved parties including individuals, healthcare professionals, government and industry [58].

## Future prospects

There are numerous unanswered questions associated with SARS-CoV-2 immunity, specifically the protective immunity. There is a necessity for different types of vaccines for differing populations such as infants and children, pregnant women, immunocompromised individuals, as a majority of the vaccines under development are targeting the healthy population i.e., 18–55 years old adults. A safe regulatory pathway must also be delineated for use of these vaccines in children, pregnant women, and immunocompromised individuals. Recent outbreaks of pertussis and measles in countries where these diseases were formerly controlled demonstrated that the success of immunization programs cannot be taken for granted. Changes that occur over decades, such as lessened compliance with immunization or modifying epidemiology of disease can overturn original assumptions about the impact of the vaccine [58]. Post-marketing surveillance should also be continued to record adverse events [65].

In order to develop a safe and effective vaccine, it is vital that pre-clinical trials are done with caution to avoid severe adverse events. Moreover, cooperation between international organizations such as the WHO, CEPI, GAVI and Bill and Melinda Gates Foundation is needed to ensure ample funding for vaccines. It is anticipated that vaccines will be available worldwide by mid-2021 to mitigate this pandemic. However, the efficacy of approved vaccines on the new mutant strains found in the United Kingdom and South Africa, are yet to be studied. The implementation of the first‐generation vaccines could be achieved by pushing the nucleic acid‐based priming vaccines followed by a booster dose of protein‐based vaccines to rein in the mortality among high‐risk communities. In parallel, more potent and efficient second‐generation vaccines can be developed and manufactured to combat mutations in the virus.

## Abbreviations

SARS-Cov-2: Severe acute respiratory syndrome coronavirus 2
Covid-19: Coronavirus disease 2019
WHO: World Health Organization
UK: United Kingdom
USA: United States of America
CVCs: COVID-19 vaccine candidate:
EUA: Emergency Use Authorization
TLRs: Toll-like receptors
FDA: Food and Drug Administration
RBD: Receptor-binding domain
TGA: Australian Therapeutic Good Administration
MHRA: The Medicines and Healthcare products Regulatory Agency
UAE: United Arab Emirates
ICMR: Indian Council of Medical Research
CDC: Centers for Disease Control and Prevention
VAERD: Vaccine-enhanced disease for inactivated vaccine candidates
VCD: Virologically confirmed dengue
DCVMN: Developing Countries Vaccine Manufacturers Network
GAVI: Global Alliance for Vaccines and Immunizations
GVAP: Global Vaccine Action Plan
CEPI: Coalition for Epidemic Preparedness Innovations
COVAX: COVID-19 Vaccine Global Access Facility
